# The cervico‐vaginal DNA methylation WID‐qEC test: An epigenetic marker associated with ovarian cancer in the absence of endometrial and cervical cancer

**DOI:** 10.1002/ijc.70354

**Published:** 2026-01-29

**Authors:** Elisa Redl, Chiara Herzog, Charlotte Vavourakis, James Barrett, Allison Jones, Iona Evans, Daniel Reisel, Ranjit Manchanda, Line Bjørge, Michal Zikan, David Cibula, Twana Alkasalias, Angelique Flöter Rådestad, Kristina Gemzell‐Danielsson, Louis Dubeau, Nicola MacDonald, Davor Jurkovic, Nora Pashayan, Martin Widschwendter

**Affiliations:** ^1^ European Translational Oncology Prevention and Screening (EUTOPS) Institute Universität Innsbruck Hall in Tirol Austria; ^2^ Research Institute for Biomedical Aging Research Universität Innsbruck Innsbruck Austria; ^3^ Department of Women's Cancer, UCL EGA Institute for Women's Health University College London London UK; ^4^ Department of Gynaecological Oncology Barts Health NHS Trust London UK; ^5^ Wolfson Institute of Population Health Queen Mary University of London London UK; ^6^ Department of Health Services Research, Faculty of Public Health & Policy London School of Hygiene & Tropical Medicine London UK; ^7^ Department of Obstetrics and Gynaecology Haukeland University Hospital Bergen Norway; ^8^ Centre for Cancer Biomarkers CCBIO, Department of Clinical Science University of Bergen Bergen Norway; ^9^ Department of Obstetrics and Gynecology Bulovka University Hospital Prague Czech Republic; ^10^ Department of Obstetrics and Gynecology, General University Hospital in Prague, First Faculty of Medicine Charles University Czech Republic; ^11^ Department of Women's and Children's Health Karolinska Institutet Stockholm Sweden; ^12^ General Directorate of Scientific Research Centre Salahaddin University‐Erbil Erbil Iraq; ^13^ Department of Pathology, Keck School of Medicine, USC/Norris Comprehensive Cancer Centre University of Southern California Los Angeles California USA; ^14^ Department of Gynaecological Oncology University College London Hospitals London UK; ^15^ Department of Public Health and Primary Care University of Cambridge Cambridge UK; ^16^ Department of Obstetrics and Gynaecology Tirol Kliniken Hall in Tirol Austria

**Keywords:** cervico‐vaginal sample; WID‐qEC test, DNA methylation, ovarian cancer

## Abstract

The DNA methylation‐based WID‐qEC test, applied to cervico‐vaginal samples, has been validated for the accurate detection of endometrial and cervical cancers. However, a small proportion of women test positive despite the absence of these cancers. The aim of this study was to explore the biological and clinical characteristics associated with such WID‐qEC‐positive cases to inform potential follow‐up strategies. We analyzed 1269 cervico‐vaginal samples from women without endometrial or cervical cancer, including healthy controls (*n* = 624), women with benign gynecological conditions (*n* = 324), and ovarian cancer cases (*n* = 321). Of the 80 WID‐qEC‐positive results, 43 (54%) were from women with ovarian cancer. WID‐qEC positivity was associated with the presence of ovarian cancer (adjusted odds ratio [OR] 2.93; 95% CI 1.75–4.95) and with a higher number of lifetime ovulatory cycles (adjusted OR 2.67; 95% CI 1.06–7.50), a known ovarian cancer risk factor. Both associations were independent of age, menopausal status, hormone replacement therapy usage, or family history of breast or ovarian cancer. Our findings suggest that in the absence of endometrial or cervical cancer, WID‐qEC positivity may indicate an elevated risk or presence of ovarian cancer. While the standalone positive predictive value (PPV) for ovarian cancer detection remains low in the general population, we outline how WID‐qEC could be used in a two‐step triage approach. In women presenting with abnormal bleeding, combining WID‐qEC positivity with a highly specific plasma‐based cell‐free DNA methylation test (e.g., with 60%–80% sensitivity and ~98.4% specificity) could theoretically yield a PPV of around 30%–40%. This hypothetical modeling is intended solely to illustrate how WID‐qEC positivity might inform future triage research, rather than to propose a clinical diagnostic algorithm.

## What's New?

The DNA methylation‐based WID‐qEC test has been validated for the accurate detection of endometrial and cervical cancers using cervico‐vaginal samples. However, a small proportion of women test positive despite the absence of these cancers. This study explored the associated biological and clinical characteristics to inform potential follow‐up strategies. The results showed that in the absence of endometrial or cervical cancer, WID‐qEC test positivity may indicate an elevated risk or the presence of ovarian cancer. While the standalone positive predictive value for ovarian cancer detection remains low in the general population, WID‐qEC could potentially be used in future two‐step triage approaches.

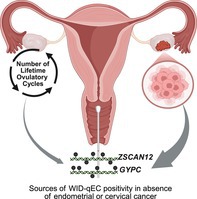

**Abbreviations**
AUB: abnormal uterine bleeding
AUC: area under the curve
BMI: body mass index
CC: cervical cancer
CI: confidence interval
Copan: FLOQswab combined with eNAT collection fluid
EC: endometrial cancer
FORECEE: Female cancer predictiOn using ceRvical omics to individualisE sCreEning and prEvention
ML: MethyLight
OC: ovarian cancer
OR: odds ratio
PMR: percentage of fully methylated reference
PPV: positive predictive value
QC: quality control
RT: room temperature
SIR: standardized incidence ratio
ThinPrep: Cervex brush combined with PreservCyt collection fluid
WID‐qEC: women's cancer risk identification—quantitative polymerase chain reaction test for endometrial cancer

## 1 INTRODUCTION

The Women's Cancer Risk Identification – quantitative polymerase chain reaction test for endometrial cancer (WID‐qEC) is a molecular assay that evaluates DNA methylation of the *ZSCAN12* and *GYPC* genes.^1^, ^2^, ^3^, ^4^, ^5^ The WID‐qEC test utilizes PCR reactions against the fully methylated alleles of CpG island regions within these two genes, with primers and probes covering 10 and 8 cytosines within the CpG dinucleotide context, respectively. The summed percentage of fully methylated reference (∑PMR) values for both genes determines the test result, with a ∑PMR ≥0.3 considered positive.^3^, ^4^, ^5^ The WID‐qEC test has demonstrated 91%–100% sensitivity for detecting endometrial and cervical cancers,^1^, ^2^, ^3^, ^4^, ^5^ with a reported false‐positive rate of 2.7% in a prospective cohort of 399 women ≥45 years of age with abnormal uterine bleeding (AUB).^3^ In the same prospective study, endometrial thickness of >3 mm measured with transvaginal ultrasound had a false‐positive rate of 54.2%.^3^

The WID‐qEC test is currently offered to women aged 45 years and older who present with AUB or postmenopausal bleeding (PMB), to identify those at highest risk of endometrial cancer and prioritize them for urgent hysteroscopy and curettage. Effective clinical implementation of the WID‐qEC test will benefit from clear guidance on managing positive test results when neither endometrial nor cervical cancer is detected.

A non‐negligible subset of women diagnosed with ovarian cancer present with AUB: In a case–control study of ovarian carcinoma, 13% and 15% of women with localized and regional disease reported AUB in the year leading up to diagnosis, making it one of the most predictive symptoms alongside abdominal pain and palpable mass.^6^ Among 43,756 Danish women with PMB, the standardized incidence ratio (SIR) comparing the observed ovarian cancer incidence with that expected in the general population was highly elevated during 0–3 months (SIR = 21.17 [95% CI: 22.47–32.56]) after hospital presentation and remained high in the following 3–12 months (SIR = 3.91 [95% CI: 2.88–5.18]) and 1–5 years (SIR = 1.45 [95% CI: 1.14–1.80]).^7^ Given these observations, we sought to explore whether WID‐qEC positivity in the absence of endometrial or cervical cancer might correlate with ovarian cancer presence or risk factors such as lifetime ovulatory cycles. This exploratory work was not designed to evaluate the WID‐qEC test as a diagnostic tool for ovarian cancer.

## 2 MATERIALS AND METHODS

Here, we analyzed the WID‐qEC test in 1269 cervico‐vaginal samples from women *without* endometrial or cervical cancer. Women were recruited as part of the FORECEE (Female cancer predictiOn using ceRvical omics to individualisE sCreEning and prEvention) programme,^8^ which aimed to develop predictive tests for breast,^9^ ovarian,^10^ and endometrial^11^ cancers using cervico‐vaginal samples. In total, the sample set included 624 controls from women in cervical screening programs; 324 controls from women with benign/borderline conditions (48 endometriosis/adenomyosis, 72 uterine fibroids, 123 benign adnexal masses, and 81 borderline tumors); and 321 samples from women newly diagnosed with ovarian cancer and prior to any treatment (217 high‐grade serous, 24 low‐grade serous, 16 mucinous, 19 endometrioid, 24 clear cell ovarian cancers, 5 ovarian carcinosarcomas, 14 non‐epithelial malignant tumors, and 2 other malignant ovarian tumors).

**FIGURE 1:**
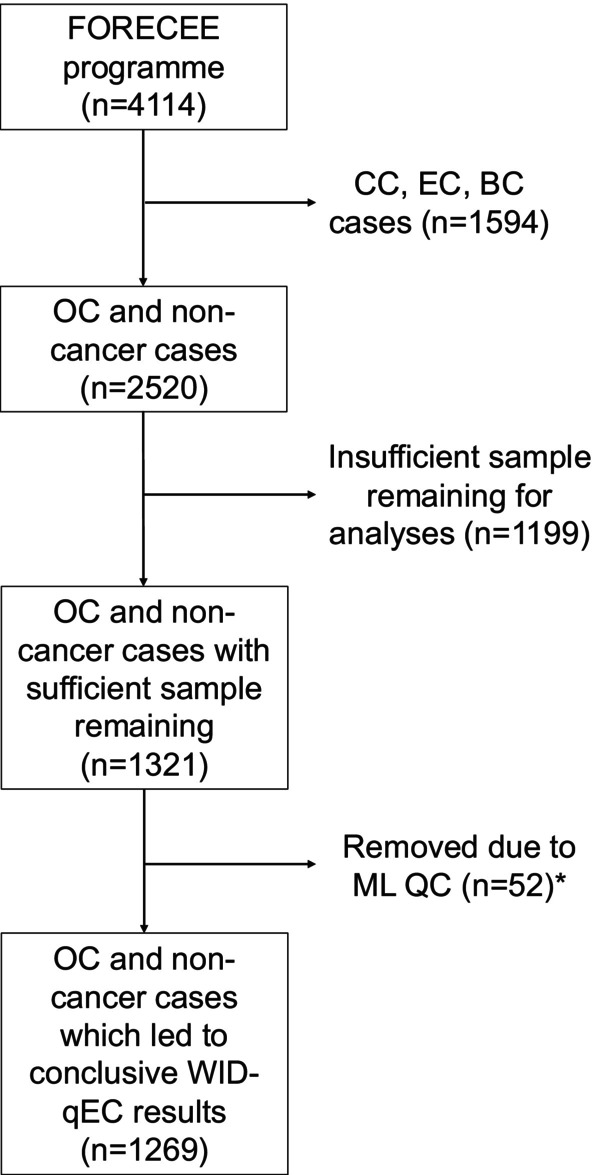
Composition of the study population with exclusion criteria. *WID‐qEC was attempted but insufficient DNA was available to reach a conclusive result. BC, breast cancer; CC, cervical cancer; EC, endometrial cancer; ML, MethyLight; OC, ovarian cancer; QC, quality control.

Cervico‐vaginal samples were collected using Cervex brushes (Rovers Medical Devices) and preserved in ThinPrep™/PreservCyt™ (Hologic) solution. Only samples with sufficient DNA remaining in the FORECEE biobank were included (Figure 1).

After bisulfite conversion of the DNA, both target regions (*ZSCAN12* and *GYPC*) and a reference region (*COL2A1*)—which lacks CpG sites and serves as a human DNA input control and a bisulfite conversion efficiency control—were amplified using one duplex and one singleplex qPCR reaction. Samples were analyzed in technical replicates, and the test result was expressed as the ∑PMR value, calculated as previously described.^3^, ^4^, ^5^ Lifetime ovulatory cycle numbers were determined by calculating the number of months between menarche and the last menstrual cycle, subtracting months of pregnancy and oral contraceptive use. All data are available in Table S1.

## 3 RESULTS AND DISCUSSION

A total of 6.3% (80/1269) of samples were WID‐qEC positive (Table 1), This includes 2.9% (18/624) of women in cervical screening programs, 5.9% (19/324) of women with benign conditions or borderline tumors, and 13.4% (43/321) of women with malignant ovarian tumors (Table 2). The vast majority of ovarian cancers (300 out of 321; 93.5%) were classified as serous, mucinous, endometrioid, or clear cell subtypes.

**TABLE 1:** Associations between reproductive and other factors in women with and without a positive WID‐qEC test.

	Total *N* = 1269	WID‐qEC negative^a^ *N* = 1189, *n* (%)	WID‐qEC positive^b^ *N* = 80, *n* (%)	Odds ratio	*p* value	Odds ratio (adjusted)	Adjusted *p* value (adjusted)
Age	1269	51.2 (20.1)^c^	57.8 (14.8)^c^	**1.04 (1.02–1.06)**	**0.000**	**1.04 (1–1.07)**	**0.028**
BMI (kg/m^2^)							
Healthy (18.5–24.9)	631	596 (50.13)	35 (43.75)	1 (reference)	—	1 (reference)	—
Underweight (<18.5)	50	47 (3.95)	3 (3.75)	1.09 (0.26–3.17)	0.893	1.16 (0.26–3.61)	0.816
Overweight (25–29.9)	344	320 (26.91)	24 (30)	1.28 (0.74–2.17)	0.372	1.18 (0.67–2.05)	0.569
Obese (>30)	239	222 (18.67)	17 (21.25)	1.3 (0.7–2.34)	0.386	1.16 (0.61–2.13)	0.636
Missing or unknown	5	4 (0.34)	1 (1.25)	—	—	—	—
Menopausal status							
Premenopausal	598	570 (47.94)	28 (35)	1 (reference)	—	1 (reference)	—
Postmenopausal	659	608 (51.14)	51 (63.75)	**1.71 (1.07–2.78)**	**0.027**	0.45 (0.2–1)	0.051
Missing or unknown	12	11 (0.93)	1 (1.25)	—	—	—	—
Hormone replacement therapy							
Never	1097	1030 (86.63)	67 (83.75)	1 (reference)	—	1 (reference)	—
<5 years	106	99 (8.33)	7 (8.75)	1.09 (0.44–2.28)	0.839	1.15 (0.45–2.54)	0.749
≥5 years	61	56 (4.71)	5 (6.25)	1.37 (0.47–3.23)	0.512	0.91 (0.29–2.33)	0.852
Missing or unknown	5	4 (0.34)	1 (1.25)	—	—	—	—
First degree relatives with breast cancer							
No	1112	1035 (87.05)	77 (96.25)	1 (reference)	—	1 (reference)	—
Yes	157	154 (12.95)	3 (3.75)	**0.26 (0.06–0.71)**	**0.024**	**0.26 (0.06–0.71)**	**0.024**
First degree relatives with ovarian cancer							
No	1193	1117 (93.94)	76 (95)	1 (reference)	—	1 (reference)	—
Yes	75	71 (5.97)	4 (5)	0.83 (0.25–2.07)	0.720	0.88 (0.26–2.27)	0.816
Missing or unknown	1	1 (0.08)	0 (0)	—	—	—	—
Estimated lifetime ovulatory cycles							
<191	297	290 (24.39)	7 (8.75)	1 (reference)	—	1 (reference)	—
191–315	298	283 (23.8)	15 (18.75)	2.2 (0.91–5.82)	0.091	1.67 (0.67–4.52)	0.285
316–404	300	279 (23.47)	21 (26.25)	**3.12 (1.37–8.02)**	**0.010**	1.74 (0.7–4.8)	0.254
≥404	300	266 (22.37)	34 (42.5)	**5.3 (2.45–13.21)**	**0.000**	**2.67 (1.06–7.5)**	**0.047**
Missing or unknown	74	71 (5.97)	3 (3.75)	—	—	—	—
Current ovarian cancer							
No	948	911 (76.62)	37 (46.25)	1 (reference)	—	1 (reference)	—
Yes	321	278 (23.38)	43 (53.75)	**3.81 (2.41–6.05)**	**0.000**	**2.93 (1.75–4.95)**	**0.000**

*Note*: Adjusted values are derived from a model including all features as covariates. Family history is defined as breast and/or ovarian cancer diagnosis in parents, siblings, or offspring (first degree relatives). The bold values indicate results with significant p‐values.

Abbreviations: 95% CI, 95% confidence interval; BMI, body mass index; OR, odds ratio.

^a^ ∑PMR <0.3.

^b^ ∑PMR ≥0.3.

^c^ Median (IQR).

**TABLE 2:** Associations between reproductive and other factors in women with and without an ovarian cancer.

	Total *N* = 1269	Control *N* = 948, *n* (%)	Cancer *N* = 321, *n* (%)	Odds ratio	*p* value	Odds ratio (adjusted)	Adjusted *p* value (adjusted)
Age	1269	49.8 (18.3)^a^	59.9 (17.1)^a^	**1.06 (1.05–1.07)**	**0.000**	**1.03 (1.01–1.05)**	**0.001**
BMI (kg/m^2^)							
Healthy (18.5–24.9)	631	487 (51.37)	144 (44.86)	1 (reference)	—	1 (reference)	—
Underweight (<18.5)	50	33 (3.48)	17 (5.3)	1.74 (0.92–3.18)	0.076	**2.8 (1.35–5.64)**	**0.004**
Overweight (25–29.9)	344	257 (27.11)	87 (27.1)	1.14 (0.84–1.55)	0.386	0.93 (0.66–1.3)	0.679
Obese (>30)	239	170 (17.93)	69 (21.5)	1.37 (0.98–1.92)	0.065	1 (0.69–1.44)	0.990
Missing or unknown	5	1 (0.11)	4 (1.25)	—	—	—	—
Menopausal status							
Premenopausal	598	529 (55.8)	69 (21.5)	1 (reference)	—	1 (reference)	—
Postmenopausal	659	411 (43.35)	248 (77.26)	**4.63 (3.46–6.26)**	**0.000**	**2.18 (1.39–3.44)**	**0.001**
Missing or unknown	12	8 (0.84)	4 (1.25)	—	—	—	—
Hormone replacement therapy							
Never	1097	824 (86.92)	273 (85.05)	1 (reference)	—	1 (reference)	—
<5 years	106	86 (9.07)	20 (6.23)	0.7 (0.41–1.14)	0.170	**0.37 (0.21–0.63)**	**0.000**
≥5 years	61	37 (3.9)	24 (7.48)	**1.96 (1.14–3.31)**	**0.013**	0.88 (0.48–1.57)	0.658
Missing or unknown	5	1 (0.11)	4 (1.25)	—	—	—	—
First degree relatives with breast cancer							
No	1112	829 (87.45)	283 (88.16)	1 (reference)	—	1 (reference)	—
Yes	157	119 (12.55)	38 (11.84)	0.94 (0.63–1.37)	0.737	0.83 (0.54–1.26)	0.391
First degree relatives with ovarian cancer							
No	1193	893 (94.2)	300 (93.46)	1 (reference)	—	1 (reference)	—
Yes	75	54 (5.7)	21 (6.54)	1.16 (0.67–1.92)	0.582	1.07 (0.6–1.86)	0.814
Missing or unknown	1	1 (0.11)	0 (0)	—	—	—	—
Estimated lifetime ovulatory cycles							
<191	297	272 (28.69)	25 (7.79)	1 (reference)	—	1 (reference)	—
191–315	298	246 (25.95)	52 (16.2)	**2.3 (1.4–3.87)**	**0.001**	1.68 (1–2.9)	0.055
316–404	300	205 (21.62)	95 (29.6)	**5.04 (3.18–8.27)**	**0.000**	**2.64 (1.59–4.52)**	**0.000**
≥404	300	178 (18.78)	122 (38.01)	**7.46 (4.73–12.16)**	**0.000**	**2.76 (1.63–4.79)**	**0.000**
Missing or unknown	74	47 (4.96)	27 (8.41)	—	—	—	—
WID‐qEC							
Negative	1189	911 (96.1)	278 (86.6)	1 (reference)	—	1 (reference)	—
Positive	80	37 (3.9)	43 (13.4)	**3.81 (2.41–6.05)**	**0.000**	**2.95 (1.78–4.93)**	**0.000**

*Note*: Adjusted values are derived from a model including all features as covariates. Family history is defined as breast and/or ovarian cancer diagnosis in parents, siblings, or offspring (first degree relatives). The bold values indicate results with significant *p*‐values.

Abbreviations: 95% CI, 95% confidence interval; BMI, body mass index; OR, odds ratio.

^a^ Median (IQR).

Age, menopausal status, family history of breast cancer, lifetime ovulatory cycles, and the presence of ovarian cancer were associated with WID‐qEC positivity in an unadjusted logistic regression analysis. After adjusting for all factors, only age (adjusted OR: 1.04; 95% CI: 1.00–1.07), family history of breast cancer (adjusted OR: 0.26; 95% CI: 0.06–0.71), the top quartile of lifetime ovulatory cycles (adjusted OR: 2.67; 95% CI: 1.06–7.50), and ovarian cancer presence (adjusted OR: 2.93; 95% CI: 1.75–4.95) remained significant. Neither body mass index (BMI) nor hormone replacement therapy was significantly associated with WID‐qEC positivity (Table 1). Overall, the presence of ovarian cancer was the strongest predictor of a WID‐qEC positive result (54% [43/80] and adjusted OR 2.95; 95% CI: 1.78–4.93; Table 2).

Among patients with epithelial ovarian cancer, cervico‐vaginal WID‐qEC positivity was observed in 13.3% (32/241) of serous, 12.5% (2/16) of mucinous, 10.5% (2/19) of endometrioid, and 16.7% (4/24) of clear cell subtypes.

The WID‐qEC test was developed for the early detection of endometrial cancer, where aberrant DNA methylation is predominantly present in cancerous compared to normal endometrial tissue.^1^ Hence, WID‐qEC positivity in a patient with ovarian cancer could suggest tumor DNA drainage via the fallopian tubes and uterus, as previously proposed by researchers assessing somatic genetic alterations.^12^, ^13^ However, given that the number of lifetime ovulatory cycles, a well‐established ovarian cancer risk factor,^14^, ^15^, ^16^, ^17^ remained an independent predictor of WID‐qEC positivity, even after adjusting for ovarian cancer presence and other covariates, we cannot exclude the possibility that WID‐qEC positivity may reflect underlying ovarian cancer risk rather than merely the presence of tumor‐derived DNA. This raises the hypothesis that WID‐qEC may act as a biological readout of cumulative ovulatory exposure in women predisposed to develop ovarian cancer.

In summary, we found that WID‐qEC positivity, in the absence of endometrial or cervical cancer, serves as a biological readout of lifetime ovulatory cycles and independently detects or predicts ovarian cancer. Because this study used a case–control design enriched for ovarian cancer, the observed associations cannot be extrapolated to population‐level performance or clinical screening metrics.

The study's limitations include incomplete information on menopausal status and lifetime ovulatory cycles, as well as the inherent challenges associated with case–control study designs. Additional limitations include the absence of direct comparison with serum biomarkers such as CA‐125 or HE4, which was beyond the scope of this study focused on understanding WID‐qEC false positives. Future studies could explore these relationships where appropriate.

The following calculation is presented purely to illustrate theoretical implications for research. It does not represent a clinical recommendation, and future modeling or prospective validation would be required to determine any practical triage value. Based on a nationwide Danish cohort study, the annual prevalence of ovarian cancer in women with PMB is 0.38%.^7^ While we did not record symptoms in the FORECEE study, under the assumption that the sensitivity of 13.4% and 3.9% false positive rate of the WID‐qEC test described in Table 2 apply to a hypothetical population of 10,000 women with PMB (38 OCs, 9962 controls), this would yield 5.1 true positives (38 × 13.4%) and 388.5 false positives (9962 × 3.9%), with a positive predictive value (PPV) of 1.29%. Although this PPV would not justify diagnostic procedures, women presenting with AUB and a positive WID‐qEC in the absence of endometrial or cervical cancer could be recommended additional triage with plasma‐based cell‐free DNA methylation,^18^ or any other test with an extremely high specificity.^19^, ^20^ In a symptomatic population, the Galleri® test for instance has a sensitivity of 64.3% and a specificity of 98.4%,^18^ and will refine true positives to 3.3 (5.1 × 64.3%) and false positives to 6.2 (388.5 × 1.6%), resulting in a PPV of 34.5%. This would suggest that in women presenting with AUB that have both a positive WID‐qEC test and a positive plasma cell‐free DNAme test, around three patients have to undergo an intraperitoneal procedure to detect one ovarian cancer.

Future studies utilizing cohort‐based cervical biobanks with long‐term follow‐up will be necessary to determine whether the WID‐qEC test appears to reflect biological correlates of lifetime ovulatory cycles and is independently associated with the presence of ovarian cancer. Further research is needed to determine whether this association could have predictive relevance.

## AUTHOR CONTRIBUTIONS

**Elisa Redl:** Investigation; writing – review and editing; formal analysis; methodology; project administration. **Chiara Herzog:** Investigation; formal analysis; data curation; writing – review and editing. **Charlotte Vavourakis:** Writing – review and editing; visualization; investigation; data curation; validation. **James Barrett:** Investigation; supervision; data curation; writing – review and editing. **Allison Jones:** Writing – review and editing; investigation; data curation; project administration. **Iona Evans:** Investigation; writing – review and editing; data curation; project administration. **Daniel Reisel:** Writing – review and editing; data curation. **Ranjit Manchanda:** Writing – review and editing; investigation; resources. **Line Bjørge:** Investigation; writing – review and editing; resources. **Michal Zikan:** Writing – review and editing; investigation; data curation; resources. **David Cibula:** Resources; data curation; writing – review and editing; investigation. **Twana Alkasalias:** Writing – review and editing. **Angelique Flöter Rådestad:** Writing – review and editing; investigation; resources. **Kristina Gemzell‐Danielsson:** Investigation; writing – review and editing; resources. **Louis Dubeau:** Writing – review and editing; investigation. **Nicola MacDonald:** Investigation; writing – review and editing; resources. **Davor Jurkovic:** Writing – review and editing; investigation; resources. **Nora Pashayan:** Resources; writing – review and editing; investigation. **Martin Widschwendter:** Conceptualization; investigation; funding acquisition; writing – original draft; supervision; resources.

## FUNDING INFORMATION

This work received funding from the European Union's Horizon 2020 research and innovation program under grant agreement No 634570 (FORECEE) and the European Union's Horizon 2020 European Research Council under grant agreement No 742432 (BRCA‐ERC); The Eve Appeal and the Land Tirol and the Standortagentur Tirol.

## CONFLICT OF INTEREST STATEMENT

E.R., C.H., J.B., A.J., I.E., and M.W. are inventors of WID‐qEC‐related patent applications. C.H., J.B., and M.W. are shareholders of Sola Diagnostics GmbH. Sola Diagnostics GmbH holds an exclusive licence to the intellectual property that protects the commercialization of the WID‐qEC test. R.M. has research grant funding for ovarian cancer surveillance/prevention from Barts Charity, Yorkshire Cancer Research, Rosetrees Trust, GSK and North East London Cancer Alliance outside this work and received honoraria for advisory board membership or lectures from Astra Zeneca/MSD/GSK/EGL. All the other authors do not have a conflict of interest.

## ETHICS STATEMENT

This study is a sub‐study within the FORECEE program, which received ethical approval from the UK Health Research Authority (REC 14/LO/1633). All participants provided written informed consent.

## Supporting information


**Table S1.** All relevant data supporting this work on cervico‐vaginal samples.

## Data Availability

All data supporting the findings of this study are available in Table S1. Further information is available from the corresponding author upon request.
